# Association of cord blood chemokines and other biomarkers with neonatal complications following intrauterine inflammation

**DOI:** 10.1371/journal.pone.0175082

**Published:** 2017-05-22

**Authors:** Yoshikazu Otsubo, Kunio Hashimoto, Taro Kanbe, Muneichiro Sumi, Hiroyuki Moriuchi

**Affiliations:** 1 Department of Pediatrics, Sasebo City General Hospital, Sasebo City, Nagasaki, Japan; 2 Department of Pediatrics, Nagasaki University Graduate School of Biomedical Sciences, Nagasaki City, Nagasaki, Japan; Johns Hopkins University, UNITED STATES

## Abstract

**Background:**

Intrauterine inflammation has been associated with preterm birth and neonatal complications. Few reports have comprehensively investigated multiple cytokine profiles in cord blood and precisely identified surrogate markers for intrauterine inflammation.

**Aim:**

To identify the cytokines and surrogate markers associated with intrauterine inflammation and subsequent neonatal complications.

**Patients and methods:**

We analyzed cord blood samples from 135 patients admitted to the neonatal intensive care unit at Sasebo City General Hospital. We retrospectively determined the associations between the presence of neonatal complications and cord blood cytokines, prenatal factors, and laboratory data at birth. A total of 27 cytokines in the cord blood were measured using a bead-based array sandwich immunoassay.

**Results:**

Both Th1 and Th2 cytokine levels were low, whereas the levels of growth factors and chemokines were high. In particular, chemokines IL-8, MCP-1, and MIP-1α were significantly higher in very premature neonates when compared with more mature neonates. In addition, some have been shown to be associated with multiple neonatal complications, including patent ductus arteriosus (PDA), respiratory distress syndrome (RDS), and chronic lung disease (CLD). Similarly, the levels of N-terminal pro-brain natriuretic peptide, nucleated RBC, and urinary β2-microglobulin were associated with these complications and chemokine levels.

**Conclusions:**

Our results suggest the association of inflammatory chemokines IL-8, MCP-1, and MIP-1α with intrauterine inflammation, premature birth, and neonatal complications in these perinatal subjects. Furthermore, the association of the aforementioned biomarkers with PDA, RDS, and CLD may help establish early diagnostic measures to predict such neonatal complications following intrauterine inflammation.

## Introduction

The exposure of offspring, especially a preterm fetus, to an adverse intrauterine environment is associated with neonatal complications in the central nervous, respiratory, and circulatory systems [1, 2]. However, the impact of intrauterine inflammation with infectious (chorioamnionitis, CAM) and non-infectious (subchorionic hematoma, SCH) etiologies on neonatal complications remains controversial. This type of inflammation is associated with brain damage and developmental problems in infants, but it may also protect against neonatal respiratory distress syndrome (RDS). The role of intrauterine inflammation in the development of neonatal chronic lung disease (CLD) is also under debate [3]. A number of studies focused on cytokines as key players in intrauterine inflammation and subsequent neonatal complications. The measurement of cytokine concentrations in the amniotic fluid, cord blood, and neonatal peripheral blood may show the extent of inflammation [4–6] and help predict neonatal complications; however, the complete and complicated cytokine network involved in disease pathogenesis cannot be understood by simply measuring cytokines of interest. Cord blood can be obtained noninvasively immediately after birth; therefore, we attempted to assess accurately the intrauterine environment by immediately and comprehensively analyzing cord blood cytokines.

In this study, cord blood samples from neonates delivered at postmenstrual ages of 23–41 weeks were comprehensively analyzed to determine their cytokine profiles. We identified associations among the presence of neonatal complications, the cytokine profiles in cord blood, and their surrogate markers.

## Materials and methods

### Subjects

Among 153 neonates admitted to our neonatal intensive care unit (NICU) from October 2012 to October 2014, we enrolled 135 cases and analyzed their cord blood specimens. Nine neonates with chromosomal abnormalities or congenital malformations were excluded from this study, and another nine, including five born by Caesarean section (C/S) and four born vaginally, were excluded due to insufficient quantity or quality of cord blood specimens. The 135 subjects were classified into three groups based on their postmenstrual ages (Group I: < 32 weeks, Group II: 32–36 weeks, and Group III: 37–41 weeks). We retrospectively reviewed the patient charts to identify any associations between the presence of neonatal complications and cord blood cytokines, prenatal factors, and laboratory data obtained immediately after birth.

### Prenatal data

Maternal data were retrospectively retrieved from maternal medical records. These data included the presence of pregnancy-induced hypertension (PIH), preterm premature rupture of membrane (PPROM), histological CAM (h-CAM), and SCH. PIH was defined as a blood pressure exceeding 140/90 mmHg and the presence of associated clinical findings. PPROM was defined as a membrane rupture prior to the onset of labor occurring before the postmenstrual age of 37 weeks. H-CAM and SCH were confirmed by histological examinations of the placenta, which were done for all subjects regardless of the diagnosis of clinical CAM and intrauterine infection. Since all mothers clinically suspected of having CAM were treated with antibiotics, none of the placental specimens was studied microbiologically, including those clinically suspected of CAM. Therefore, we dealt with histologically confirmed CAM (h-CAM), not microbiologically confirmed CAM, in this study.

### Neonatal data

Neonatal data were retrospectively obtained from the infants’ medical records. The following neonatal factors were analyzed: small for gestational age (SGA), light for date (LFD), RDS, patent ductus arteriosus (PDA), CLD, retinopathy of prematurity (ROP), and periventricular leukomalacia (PVL). SGA was defined as both a height and weight less than the 10th percentile at birth. LFD was defined as only a weight less than the 10th percentile. Diagnoses of RDS, PDA, CLD, and ROP were given to patients requiring pulmonary surfactant therapy, those with compatible echocardiographic findings and requiring indomethacin therapy, those requiring oxygen supplementation at a corrected postmenstrual age of 36 weeks, and those requiring laser photocoagulation, respectively. PVL was diagnosed by ultrasound or magnetic resonance imaging of the brain.

### Sample collection

Immediately after delivery, we collected cord blood samples aseptically after sterilizing the puncture sites and centrifuged at 3,000 rpm for 10 minutes. Sera were isolated, preserved at -30°C, and subsequently used for the measurement of cytokines and N-terminal pro-brain natriuretic peptide (NT-proBNP). Venous blood samples were collected shortly after birth for additional blood examinations, including nucleated red blood cells (NRBC), and urine specimens were obtained within 2 days after birth for urinary β2-microgloblin (MG) measurements.

### Cytokine assay

A total of 27 cytokines were measured with a sandwich immunoassay and the bead array method Bio-Plex^™^ (Human Cytokine27-Plex Panel, Bio-Rad, Hercules, California, USA). The measured cytokines included the inflammatory cytokines tumor necrosis factor (TNF)-α, interleukin (IL)-6, IL-1β, and IL-1ra; the Th1 cytokines interferon (INF)-γ, IL-2, and IL-12 (p70); the Th2 cytokines IL-4, Il-5, IL-10, and IL-13; the Th17 cytokine IL-17; the chemokines IL-8, INF-γ-induced protein (IP)-10, monocyte chemotactic protein (MCP)-1, macrophage inflammatory protein (MIP)-1α, MIP-1β, eotaxin, and regulated on activation, normal T cell expressed and secreted (RANTES); and growth factors IL-7, IL-9, IL-15, granulocyte colony-stimulating factor (G-CSF), granulocyte macrophage colony-stimulating factor (GM-CSF), fibroblast growth factor (FGF), platelet-derived growth factor (PDGF), and vascular endothelial growth factor (VEGF).

### Statistical analyses

Group-specific test values and the frequency of complications are shown as the mean ± standard deviation (SD) or as a percentage. The cytokine data did not show a normal distribution; therefore, we have reported the log-transformed data with their median values (the 50^th^ percentile) and the lowest (the 25^th^ percentile) and highest quartile (the 75^th^ percentile). We used the Kruskal-Wallis test for comparisons between multiple groups and the Dunn’s test for multiple comparisons. We used the Mann-Whitney U test to evaluate the associations between cytokines and biomarkers (cord blood NT-proBNP, NRBC, and urinary β2-MG) with prenatal factors and neonatal complications. We used a Spearman’s rank correlation to determine any correlations between cytokines and biomarkers. Statistical significance was defined as *p* < 0.05.

### Ethics approval

This study was approved by the institutional ethics committee (Sasebo City General Hospital Ethics Committee, **Approval number**: 2010-A-27), and written informed consent was obtained from the parents of all study participants.

## Results

### Age-specific characteristics of neonatal and prenatal factors

Birth weight and Apgar scores at 1 and 5 minutes were significantly lower in Group I when compared with the other groups (Table 1). The differences in the rates of C/S, PIH, and h-CAM were not significant among the three groups, and no significant difference in the PPROM rate was observed between Groups I and II. A lower gestational age corresponded to a higher rate of SCH.

**Table 1:** Clinical characteristics of the neonatal and prenatal factors.

Characteristics	Group I Very Preterm (<32 wk)	Group II Moderately Preterm (32–36 wk)	Group III Term (37–41 wk)	P value* Group I–II–III	Dunn’s test**
N (male/female)	33 (21:12)	66 (41:25)	36 (19:17)	0.580	
Gestational age (weeks), mean ± SD	28.5 ± 2.5	34.8 ± 1.4	38.5 ± 1.4	<0.001	I–II I–III II–III
Birth weight (g), mean ± SD	1,055 ± 318	2,035 ± 386	2,513 ± 688	<0.001	I–II I–III II–III
Apgar score at 1 min, mean ± SD	5.9 ± 1.8	7.3 ± 1.6	7.6 ± 0.6	<0.001	I–II I–III
Apgar score at 5 min, mean ± SD	7.6 ± 1.2	8.7 ± 0.9	8.7 ± 0.6	<0.001	I–II I–III
Caesarean section, n (%)	25 (75.8%)	36 (54.5%)	21 (58.3%)	0.118	
PIH, n (%)^a^	6 (18.2%)	10 (15.2%)	5 (13.9%)	0.867	
Histological chorioamnionitis, n (%)	15 (45.5%)	22 (33.3%)	9 (25.0%)	0.198	
PPROM, n (%)^b^	17 (51.5%)	27 (40.9%)	0 (0%)	0.316	
Subchorionic hemorrhage, n (%)	7 (21.2%)	5 (7.6%)	1 (2.8%)	0.025	I–II I–III

* P value for the Kruskal—Wallis test and Chi-square test.

** Pairs of groups for which there are statistical differences according to Dunn’s test (significant level 0.05).

^a^ PIH: pregnancy-induced hypertension.

^b^ PPROM: preterm premature rupture of the membrane.

### Age-specific characteristics of blood and urine test results

White blood cell (WBC) counts were significantly higher in Group III, and procalcitonin (PCT) levels were significantly higher in Groups I and II. There were no significant differences in C-reactive protein (CRP), capillary blood gas pH (CBG pH), and lactate. Cord blood NT-proBNP and NRBC and urinary β2-MG levels were highest in Group I, followed by Group II (Table 2).

**Table 2:** The blood and urine analysis among the gestational-age subgroups.

	Group I Very Preterm (<32 wk)	Group II Moderately Preterm (32–36 wk)	Group III Term (37–41 wk)	P value* Group I–II–III	Dunn’s test**
WBC (/μl)^a^ (mean ± SD)	10,492 ± 8,064	11,806 ± 6,443	15,454 ± 7,043	<0.001	I–II I–III
CRP (mg/dl)^b^ (mean ± SD)	0.46 ± 0.5	0.49±1.1	0.79 ± 1.3	0.127	
PCT (ng/ml)^c^ (mean ± SD)	2.5 ± 7.5	2.6 ± 13.7	0.6 ± 1.8	<0.001	I–II I–III
CBG pH^d^ (mean ± SD)	7.26 ± 0.07	7.26 ± 0.07	7.26 ± 0.08	0.368	
Lactate (mmol/L) (mean ± SD)	3.6 ± 2.7	3.2 ± 1.4	4.1 ± 2.4	0.209	
NT proBNP (pg/ml)^e^ (mean ± SD)	7,498 ± 14,279	2,040 ± 2,626	1,088 ± 1,010	<0.001	I–II I–III
NRBC (/μl)^f^ (mean ± SD)	3,035 ± 7,895	2,613 ± 8,465	855 ± 1,104	0.007	I–III
Urine β2-MG (×10^4^μg/gCr)^g^ (mean ± SD)	9.9 ± 16.3	2.3 ± 7.1	0.7 ± 1.2	0.001	I–II I–III

* P value for the Kruskal—Wallis test.

** Pairs of groups for which there are statistical differences according to Dunn’s test (significant level 0.05).

^a^ WBC: white blood cell.

^b^ CRP: C-reactive protein.

^c^ PCT: procalcitonin.

^d^ CBG: capillary blood gas.

^e^ NT proBNP: N-terminal proB-type natriuretic protein.

^f^ NRBC: nucleated red blood cell.

^g^ β2-MG: β2-mioglobin.

### Profiles of 27 cytokines in the cord blood

**Fig 1:**
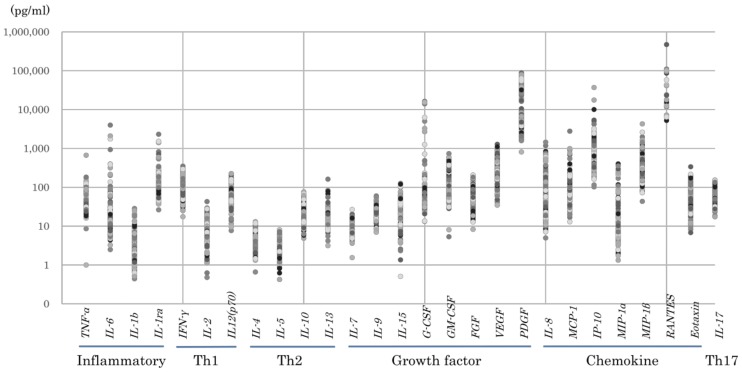
Logarithmic distribution of cytokines. High levels of inflammatory cytokines, growth factors, and chemokines as well as low levels of Th1, Th2, and Th17 cytokines were observed.

**Fig 2:**
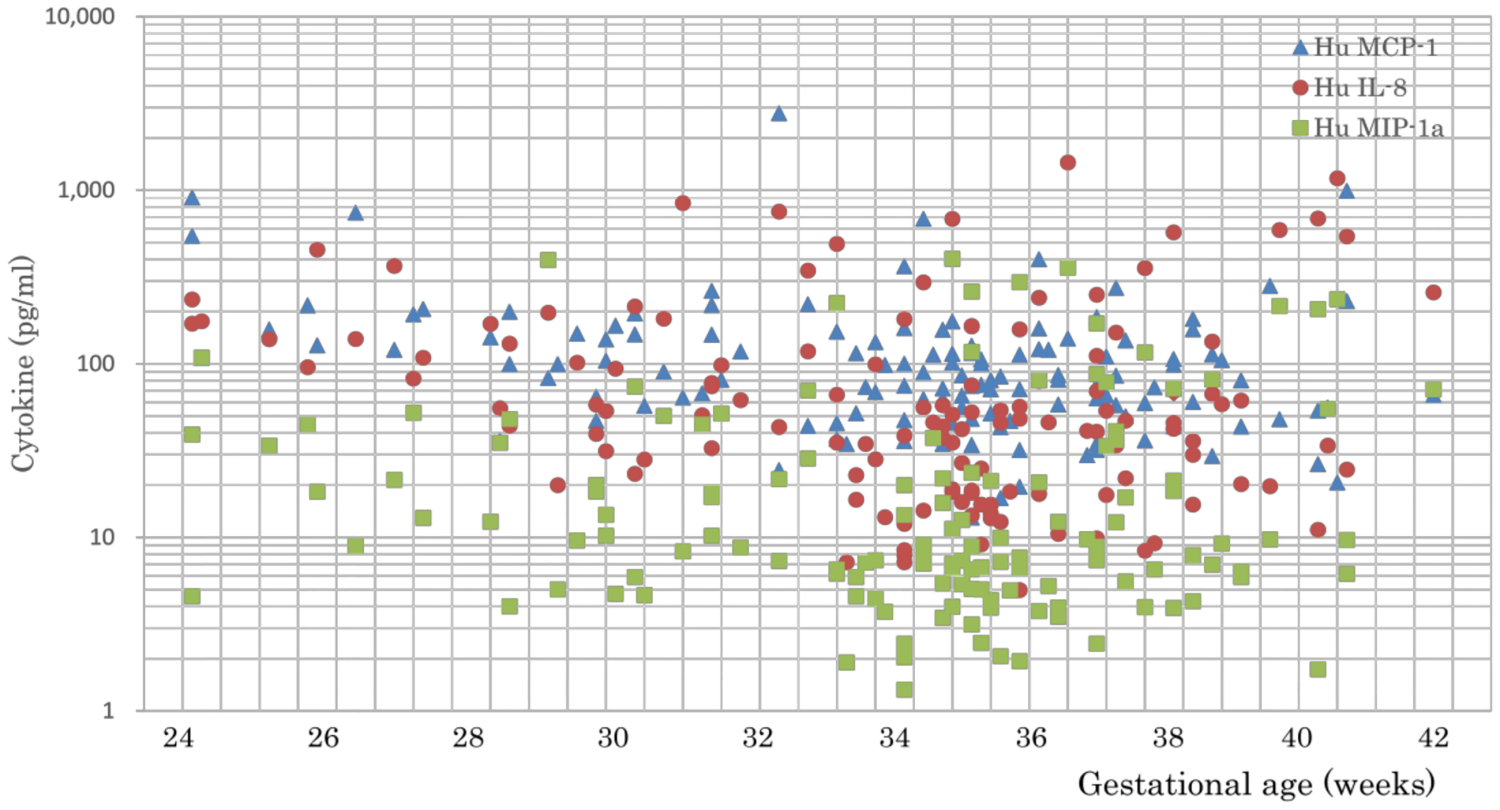
Levels of chemokines IL-8, MCP-1, and MIP-1α among age-specific groups. The levels of IL-8, MCP-1, and MIP-1α were significantly higher in younger neonates (Group I).

Fig 1 shows the logarithmic distribution of the cytokines tested in this study. Overall, the levels of inflammatory cytokines, growth factors, and chemokines were high, whereas the levels of Th1, Th2, and Th17 cytokines were low. Comparisons among age groups (Table 3) revealed that the levels of the inflammatory cytokines IL-6, IL-1β, and IL-1ra, the Th1 cytokine IL-13, and the chemokine MIP-1β were significantly higher in Group III when compared with Group II. The Th2 cytokines IL-4 and IL-10 were significantly higher in Group III when compared with Group I. The growth factor PDGF was significantly higher in Group II and Group III when compared with Group I. Eotaxin and IL-17 levels were higher in older neonates, whereas IL-8, MCP-1 and MIP-1α levels were significantly higher in younger neonates (Fig 2).

**Table 3:** Cytokine levels among gestational-age subgroups.

Cytokines	Group I Very Preterm (<32 wk) Median (25th –75th percentile)	Group II Moderately Preterm (32–36 wk) Median (25th–75th percentile)	Group III Term (37–41 wk) Median (25th–75th percentile)	P value* Group I–II–III	Dunn’s test**
TNF-α	43.2 (31.8–66.1)	35.8 (27.9–76.6)	47.9 (32.7–92.5)	0.225	
IL-6	17.0 (9.5–37.6)	11.3 (6.9–19.7)	17.3 (10.1–27.4)	0.014	II–III
IL-1b	2.5 (1.9–4.6)	1.8 (1.0–4.1)	3.0 (1.5–5.8)	0.044	II–III
IL-1ra	108.8 (66.1–190.9)	99.6 (76.4–181.0)	153.3 (103.6–214.5)	0.03	II–III
IFN-γ	77.9 (54.8–123.0)	76.3 (47.1–162.2)	83.4 (62.6–180.9)	0.504	
IL-2	9.0 (5.1–14.6)	7.5 (2.9–14.2)	7.1 (5.3–15.4)	0.607	
IL12 (p70)	67.9 (47.1–84.8)	76.6 (51.8–96.6)	79.8 (53.1–101.0)	0.124	
IL-4	3.0 (2.1–5.0)	6.0 (2.7–8.3)	6.4 (3.4–9.2)	0.005	I–III
IL-5	2.4 (1.7–3.4)	2.6 (1.7–4.3)	2.6 (1.9–4.4)	0.729	
IL-10	16.0 (11.0–22.0)	17.8 (12.2–27.7)	24.9 (13.2–32.9)	0.042	I–III
IL-13	12.3 (9.7–17.0)	13.3 (10.9–16.7)	17.7 (12.3–22.6)	0.028	II–III
IL-7	8.1 (6.7–11.1)	9.0 (6.0–11.8)	10.2 (7.3–12.3)	0.248	
IL-9	22.7 (19.6–28.3)	18.8 (14.7–23.7)	22.1 (18.4–28.8)	0.016	I–II
IL-15	22.5 (11.2–27.5)	24.5 (11.1–53.5)	22.2 (14.8–40.5)	0.531	
G-CSF	57.4 (39.6–185.3)	44.1 (33.9–71.1)	60.6 (42.1–76.2)	0.114	
GM-CSF	136.9 (90.4–191.8)	127.9 (61.8–239.1)	170.3 (83.6–283.8)	0.625	
FGF	59.9 (49.4–77.8)	60.7 (25.8–89.3)	62.0 (27.5–96.1)	0.6	
VEGF	254.5 (174.2–523.2)	249.4 (167.7–400.9)	273.7 (194.7–450.2)	0.469	
PDGF	4626.8 (2847.9–6953.5)	7775.0 (4582.3–28403.5)	7868.0 (5255.4–38789.9)	0.001	I–II, I–III
IL-8	95.5 (53.4–170.6)	35.1 (15.4–57.4)	46.4 (23.9–138.5)	<0.001	I–II
MCP-1	138.0 (90.0–194.5)	75.1 (46.8–114.6)	68.9 (49.3–110.5)	<0.001	I–II, I–III
IP-10	570.7 (327.5–1143.9)	766.2 (373.0–1249.0)	722.4 (526.7–1238.5)	0.256	
MIP-1α	18.0 (8.9–44.6)	7.0 (4.3–15.2)	10.9 (6.4–71.4)	0.002	I–II, II–III
MIP-1β	312.3 (227.4–463.5)	237.2 (152.9–387.6)	347.2 (237.9–603.6)	0.009	II–III
RANTES	12962.8 (7007.8–17701.6)	92023.7 (42499.9–105606.8)	29966.2 (16478.4–53243.2)	0.16	
Eotaxin	54.4 (27.8–101.0)	58.0 (39.4–101.5)	85.3 (67.0–113.8)	0.002	I–II, II–III
IL-17	41.0 (32.3–62.3)	63.9 (49.2–88.2)	81.3 (55.5–108.1)	<0.001	I–II, I–III

* P value for the Kruskal—Wallis test.

** Pairs of groups for which there are statistical differences according to Dunn’s test (significant level 0.05).

### Cytokine levels for different delivery modes in Group I

Table 4 compares the cytokine levels between two delivery modes (C/S and vaginal delivery) among subjects in Group I. There were no statistically significant differences in the cytokine levels between the two delivery modes.

**Table 4:** Comparison of cytokine levels between two delivery modes among subjects in Group I.

Cytokines	Caesarean section	Vaginal delivery	P value
	Median (25th-75th percentile)	Median (25th-75th percentile)	
TNF-α	43.2 (31.8–65.6)	43.2 (31.8–66.1)	0.282
IL-6	13.3 (9.2–28.2)	17.0 (9.5–37.6)	0.094
IL-1b	2.3 (1.8–4.0)	2.5 (1.9–4.6)	0.122
IL-1ra	104.6 (66.1–182.6)	108.7 (66.1–190.9)	0.132
IFN-γ	75.8 (56.4–120.8)	77.9 (54.8–123.0)	0.213
IL-2	9.0 (5.0–13.6)	9.0 (5.1–14.6)	0.551
IL12(p70)	65.8 (47.1–80.6)	67.9 (47.1–84.8)	0.541
IL-4	2.9 (2.1–5.0)	3.1 (2.1–5.1)	0.147
IL-5	2.4 (1.7–3.3)	2.5 (1.7–3.4)	0.183
IL-10	15.0 (10.7–21.9)	16.0 (11.0–22.0)	0.358
IL-13	12.6 (9.8–17.0)	12.3 (9.7–17.0)	0.097
IL-7	7.9 (6.8–11.1)	8.1 (6.7–11.1)	0.548
IL-9	22.8 (19.7–27.9)	22.7 (19.6–28.3)	0.122
IL-15	19.7 (10.2–24.8)	22.5 (11.2–27.5)	0.543
G-CSF	54.9 (39.2–101.9)	57.4 (39.6–185.3)	0.054
GM-CSF	136.2 (91.1–224.9)	136.9 (90.4–191.8)	0.417
FGF	57.6 (48.4–76.3)	59.9 (49.4–77.8)	0.531
VEGF	249.0 (175.1–452.2)	254.5 (174.2–523.2)	0.317
PDGF	4684.3 (3012.4–6920.5)	4626.8 (2847.9–6953.5)	0.535
IL-8	94.6 (51.2–162.1)	95.5 (53.4–170.6)	0.486
MCP-1	132.8 (84.5–185.7)	138.0 (90.0–194.5)	0.531
IP-10	483.7 (304.7–1034.7)	570.7 (327.5–1143.9)	0.205
MIP-1α	18.1 (9.7–44.7)	18.0 (8.9–44.6)	0.298
MIP-1β	357.9 (242.4–479.1)	312.3 (227.4–463.5)	0.173
RANTES	11689.7 (6804.5–14917.5)	12962.8 (7007.8–177701.5)	0.078
Eotaxin	55.0 (28.1–102.3)	54.4 (27.8–101.0)	0.561
IL-17	40.3 (32.8–61.3)	41.0 (32.3–62.3)	0.563

The cytokine levels showed no statistically significant differences between the two delivery modes (by Mann-Whitney U test). The data are presented as the median and interquartile range (25th-75th percentile) (significance level, 0.05).

### Relationship between neonatal complications and cytokines/biomarkers in preterm neonates

Among preterm neonates (Groups I and II), complications of RDS, PDA, and CLD positively correlated with IL-8, MCP-1, and MIP-1α levels (Table 5). In addition, preterm neonates with RDS, PDA, or CLD had significantly higher levels of cord blood NT-proBNP and NRBC and urinary β2-MG than preterm neonates without these complications. There were only six cases of ROP and no cases of PVL; thus, we were unable to conduct statistical analyses of these complications.

**Table 5:** Relationship between neonatal complications and the cytokines/biomarkers in preterm neonates.

Complications	Cytokine	Cytokine levels in the two groups with/without neonatal complications		p-value
		+	-	
RDS^a^	IL-8	138.6 (51.0–201.0)	42.0 (17.9–76.3)	<0.001
	MCP-1	152.5 (106.9–201.1)	80.9 (47.7–118.9)	<0.001
	MIP-1α	15.8 (8.4–46.5)	7.3 (4.8–20.2)	0.016
CLD^b^	IL-8	169.9 (88.8–173.1)	42.6 (18.2–94.9)	0.004
	MCP-1	192.4 (132.8–380.6)	83.6 (54.9–122.8)	0.001
	MIP-1α	21.4 (11.5–41.8)	7.5 (4.8–20.2)	0.018
PDA^c^	IL-8	138.8 (101.9–205.2)	42.6 (18.2–94.9)	<0.001
	MCP-1	192.4 (132.8–380.6)	84.9 (54.9–128.6)	0.001
	MIP-1α	33.7 (15.6–41.8)	7.5 (4.8–20.2)	0.01
	Biomarker			
RDS	NT-proBNP	2227 (1411–4746.5)	1234 (786–2518)	0.002
	NRBC	1902 (756–3242)	810 (399–1703.5)	0.033
	Urine β2-MG	13.1 (0.4–17.4)	0.71 (0.06–1.91)	<0.001
CLD	NT-proBNP	3222.5 (1726.7–4507.2)	1285.5 (842.5–2508)	0.009
	NRBC	1904 (1394.5–3126)	822.5 (403.5–1794)	0.002
	Urine β2-MG	14.9 (8.2–20.0)	0.71 (0.06–2.0)	<0.001
PDA	NT-proBNP	3222.5 (1726.7–5823.2)	1285.5 (842.5–2508)	0.004
	NRBC	1904 (1394.5–3126)	822.5 (403.5–1794)	0.02
	Urine β2-MG	17.4 (10.2–20.4)	0.71 (0.06–2.0)	<0.010

^a^ RDS: respiratory distress syndrome.

^b^ CLD: chronic lung disease.

^c^ PDA: patent ductus arteriosus.

Only the cytokine levels where the difference between the presence and absence of neonatal complications was statistically significant are shown in this Table 5. All of the biomarker levels shown above were statistically significant (according to the Mann-Whitney U test). Only preterm neonates (Groups I and II) for analysis of RDS, CLD, and PDA were used. Data are presented as the median and quartile (25th–75th percentile).

Furthermore we compared cytokine levels between premature infants (Group I) with and without neonatal complications. Among them, development of RDS was positively correlated with MCP-1 (*p* = 0.015) and IL-8 (*p* = 0.030). Development of CLD was positively correlated with MCP-1 (*p* = 0.018). And development of PDA was positively correlated with MCP-1 (*p* = 0.022) and IL-8 (*p* = 0.010).

### Relationship between biomarkers and cord blood cytokine profiles in preterm neonates

**Fig 3:**
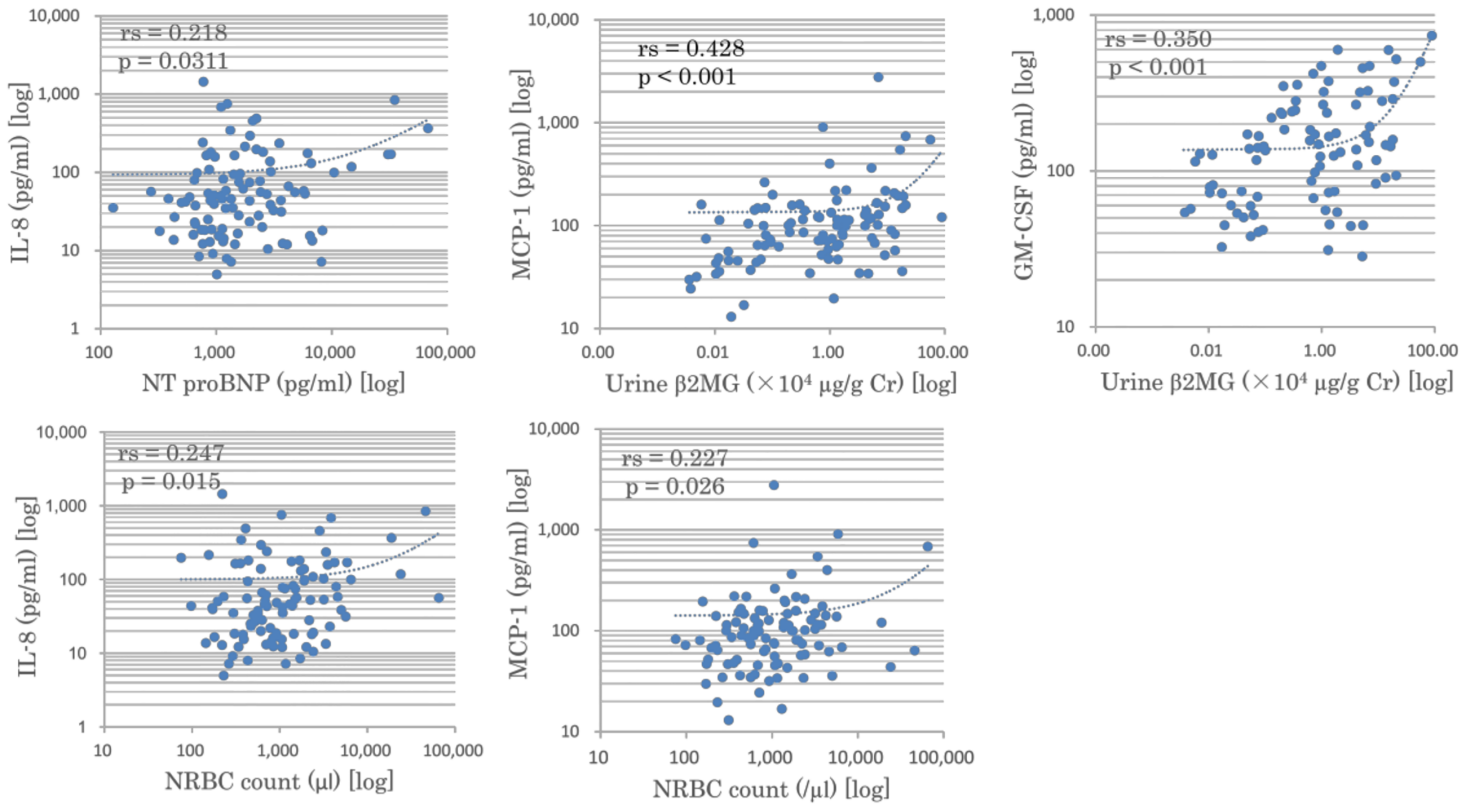
Correlations between chemokines and biomarkers. Significant positive correlations were observed between the following pairs: IL-8 and cord blood NT-proBNP (*p* = 0.031); IL-8 and NRBC (*p* = 0.015); MCP-1 and NRBC (*p* = 0.026); MCP-1 and urinary β2-MG (*p* < 0.001); and GM-CSF and urinary β2-MG (*p* < 0.001).

We analyzed the correlations between three selected biomarkers and cord blood cytokine profiles in preterm neonates (Groups I and II) by comparing all possible combinations of the 27 cytokines and 3 biomarkers (NT-proBNP, NRBC, and urinary β2-MG) (Fig 3).

## Discussion

We comprehensively analyzed 27 cytokines in cord blood specimens derived from 135 neonates who were admitted to our NICU, including 99 preterm neonates. Subjects were classified according to postmenstrual ages into Groups I (<32 weeks), II (32–36 weeks), and III (37–41 weeks). We examined the gestational age-specific characteristics as well as the relationship between cytokine profiles and neonatal complications caused by intrauterine inflammation. Furthermore, we identified surrogate markers associated with cord blood cytokines and neonatal complications.

Cytokine networks can fuel intrauterine inflammation, which often increases the risk of premature labor. In cases of infectious inflammation (e.g., CAM), macrophage activation results in the increased expression of chemokines and inflammatory cytokines and the production of prostaglandin [7]. In cases of non-infectious inflammation (e.g., SCH), thrombin induces the expression of inflammatory cytokines via the protease-activated receptor [8] and subsequent cervical ripening.

As previously reported [6], cord blood cytokine profiles showed low Th1/Th2 cytokine levels and high levels of growth factors and chemokines (Fig 1). Specifically, the chemokines IL-8, MCP-1, and MIP-1α showed significantly higher levels in very preterm neonates (Group I) when compared with more mature neonates (Fig 2) (Table 3). In addition, these chemokines showed a positive association with neonatal complications, including RDS, CLD, and PDA (Table 5). Therefore, we believe that MCP-1 and IL-8 are good candidate biomarkers for differentiating higher-risk premature infants from lower-risk ones and hope to develop practical tools useful for predicting their delivery.

We investigated several organ biomarkers for age-specific characteristics, correlations with cytokine profiles, and associations with neonatal complications. Our results showed that levels of cord blood NT-proBNP and NRBC and urinary β2-MG were the highest in very preterm neonates, followed by moderately preterm neonates (Table 2). We previously reported that cord blood NT-proBNP served as a stress marker reflecting an adverse intrauterine environment [9]. An elevated NRBC count was directly correlated with cord blood IL-6 levels in the setting of inflammation-associated preterm birth [10], and NRBC counts were higher in cases of fetal inflammatory response syndrome [11]. Furthermore, urinary β2-MG reflected intrauterine inflammation and was useful for predicting the onset of CLD [12].

Among the cytokines and organ biomarkers tested, significant correlations were observed between the following parameters: IL-8 and NT-pro BNP; IL-8 and NRBC; MCP-1 and urinary β2-MG; MCP-1 and NRBC; and GM-CSF and urinary β2-MG (Fig 3). The levels of these biomarkers positively correlated with the neonatal complications of RDS, CLD, and PDA (Table 5). Thus, these organ biomarkers are potential surrogate markers that represent the extent of intrauterine inflammation and predict neonatal complications.

Several other factors may also influence the cytokine levels in cord blood specimens. First, gender differences in the cord blood IL-6 levels [13] and predisposition to sepsis [14] have been previously reported. Although not statistically significant, the male/female ratios were slightly different among the groups in this study, with more males than females in Groups I and II (Table 1). This may have contributed to our findings for the cytokine profiles. Second, the influence of delivery mode on cytokine levels is controversial [15, 16]. C/S rates were high, especially in Group I, because emergency C/S was more often required for infants with non-reassuring situations in this group than in other groups. Such non-reassuring situations *in utero* may be associated with higher cytokine levels in the cord blood. Our study, however, showed no statistically significant difference in the cytokine levels between the C/S and vaginal delivery cases in Group I (Table 4). We do not believe there was any selection bias due to differences in the delivery modes, since only nine cases (five C/S and four vaginal deliveries) were excluded for technical reasons. Further studies in larger populations may clarify this issue.

The presence of chemokine IL-8 in the amniotic fluid, along with inflammatory cytokines, such as TNF-α, IL-6 and IL-1β, was associated with intrauterine inflammation and preterm birth [4]. Takahashi et al. reported that IL-6 and the chemokines IL-8 and MCP-1 in the cord blood were closely related to the development of neonatal complications in preterm neonates [6]. Matoba et al. reported increased levels of MCP-1, MIP-1α, and MIP-1β in the cord blood of preterm infants when compared with term infants [5]. In an experiment using preterm sheep fetuses, lipopolysaccharide-induced CAM increased the expression of MCP-1 and MCP-2, suggesting that these chemokines play a key role in fetal inflammation [17]. The BNP receptor was identified in human monocytes, and BNP treatment inhibited primary monocyte chemotaxis, possibly by antagonizing chemokine-induced inflammation [18]. In adults, IL-8 and MCP-1 are involved in chronic inflammatory conditions, such as metabolic syndrome and atherosclerosis [19]. In patients with chronic heart failure, a correlation was shown between IL-8, MCP-1, and serum BNP levels [20]. Thus, chemokines may play a critical role in chronic inflammation, and BNP may serve as a marker of not only heart failure but also chronic inflammation.

We determined that very preterm infants were likely to be exposed to chronic inflammation, beginning with the increased expression of chemokines, such as IL-8 and MCP-1. In other words, more serious intrauterine inflammation resulted in an earlier delivery and higher morbidity with neonatal complications. The measurement of cord blood cytokines, especially chemokines, as well as their surrogate markers, such as NT-proBNP and NRBC and urinary β2-MG, in preterm neonates can help determine the extent of intrauterine inflammation and predict the likelihood of neonatal complications associated with prematurity. The development of practically useful measurements may also lead to earlier therapeutic interventions. Anti-cytokine/chemokine therapy may be useful for pregnant women [21], but we should pay careful attention to its potentially detrimental effects on infection control.

In conclusion, the chemokines IL-8, MCP-1, and MIP-1α are associated with intrauterine inflammation, premature birth, and neonatal complications, implying their roles as triggers of those perinatal events. Furthermore, the biomarkers NT-pro BNP, NRBC, and β2-MG may serve as supportive markers for the early detection of neonatal complications following intrauterine inflammation in these perinatal subjects.

## Supporting information

S1 Table Incidence of neonatal complications among age-specific groups. The incidence of SGA and LFD was significantly higher in Group III, which is not surprising because these conditions were the likely reason for admission of this age group to the NICU. The incidence of RDS, PDA, CLD, and ROP, all of which accompany prematurity, were significantly higher in Group I when compared with the other groups.
(JPG)

S2 Table Relationship between prenatal factors and cord blood cytokines. Among the prenatal factors, h-CAM was associated with significantly higher levels of TNF-α, IL-6, IL-1β, IL-1ra, IFN-γ, IL-2, IL-5, IL-7, G-CSF, FGF, and IL-8. In contrast, PIH was associated with significantly higher levels of IFN-γ, IL-13, FGF, MIP-1β, and eotaxin. Neither PPROM nor SCH was related to age-specific changes in cytokine levels.
(JPG)
