# Supplementary figures and images for: Association of cord blood chemokines and other biomarkers with neonatal complications following intrauterine inflammation
Source: PLoS One. 2017 May 22;12(5):e0175082. doi: 10.1371/journal.pone.0175082 (PMC5439663; doi:10.1371/journal.pone.0175082)

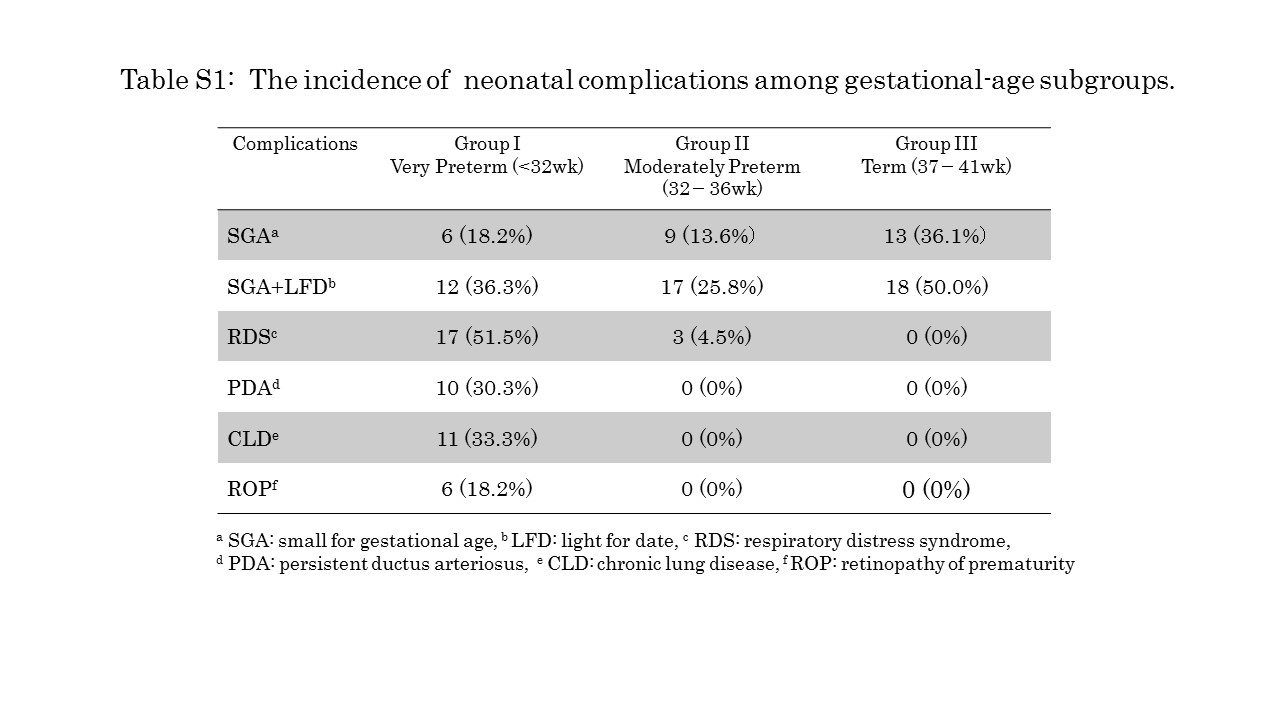

Supplement: S1 Table — The incidence of SGA and LFD was significantly higher in Group III, which is not surprising because these conditions were the likely reason for admission of this age group to the NICU. The incidence of RDS, PDA, CLD, and ROP, all of which accompany prematurity, were significantly higher in Group I when compared with the other groups. (JPG) [file pone.0175082.s001.JPG]

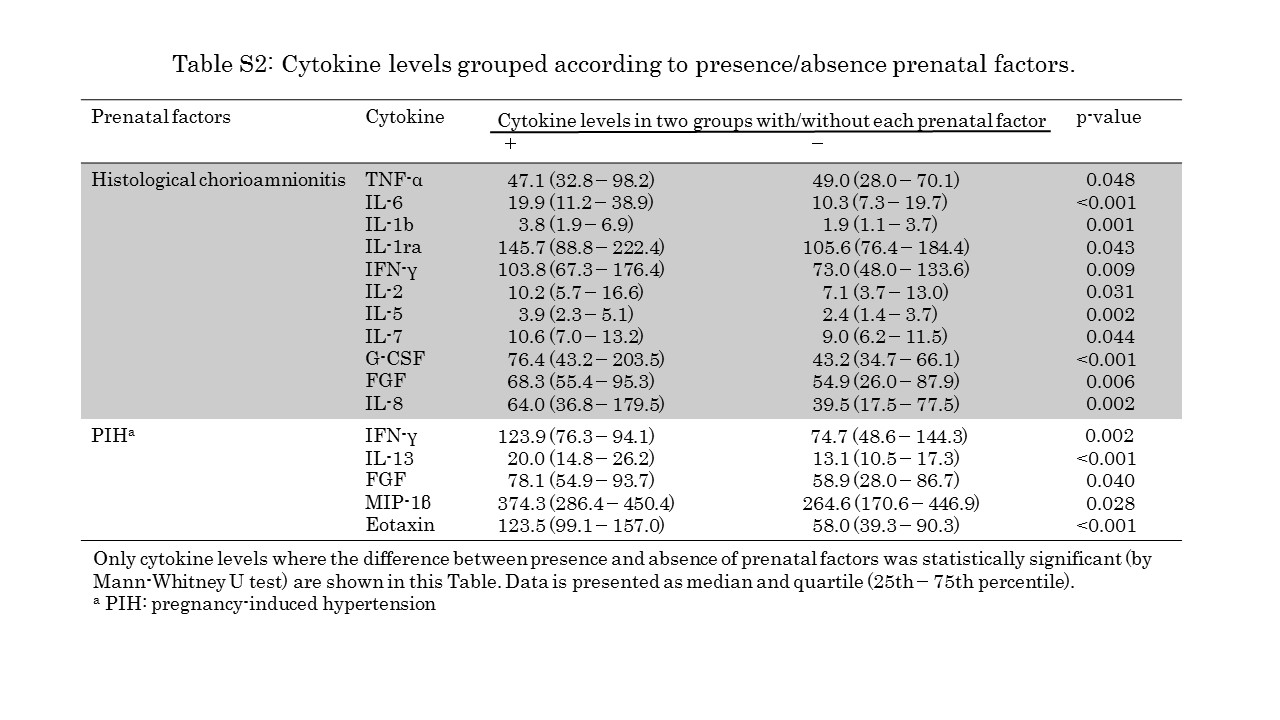

Supplement: S2 Table — Among the prenatal factors, h-CAM was associated with significantly higher levels of TNF-α, IL-6, IL-1β, IL-1ra, IFN-γ, IL-2, IL-5, IL-7, G-CSF, FGF, and IL-8. In contrast, PIH was associated with significantly higher levels of IFN-γ, IL-13, FGF, MIP-1β, and eotaxin. Neither PPROM nor SCH was related to age-specific changes in cytokine levels. (JPG) [file pone.0175082.s002.JPG]
